# Modulation of ion channels as emerging therapeutic targets in the treatment of diabetic neuropathy

**DOI:** 10.17179/excli2026-9383

**Published:** 2026-04-22

**Authors:** Tanya Gupta, Alimam Ansari, Rishabh Chalotra, Abhitinder Kumar, Thakur Gurjeet Singh, Randhir Singh

**Affiliations:** 1 Laboratory of Neuroendocrinology and Metabolic Disorders, Department of Pharmacology, Central University of Punjab, Ghudda, Bathinda, 151401, Punjab, India; 2 Centre for Research Impact & Outcome, Chitkara College of Pharmacy, Chitkara University, Rajpura, 140401, Punjab, India

**Keywords:** diabetic neuropathy, ion channel, Nav channel, Cav channel, Kv channel, TRP channels, purinergic receptors, PIEZO channels

## Abstract

Diabetic neuropathy (DN) is a prevalent microvascular complication of diabetes mellitus, characterized by hyperalgesia and allodynia that severely impair quality of life. Current treatment approaches do not provide adequate relief, largely due to the multifactorial nature of disease pathogenesis. Growing evidence indicates that dysregulation of multiple ion channel families is a central mechanism underlying sensory neuron hyperexcitability and chronic pain in DN. This review comprehensively discusses the roles of major ion channel families, including voltage-gated sodium (Naᵥ), calcium (Caᵥ), and potassium (Kᵥ) channels, transient receptor potential (TRP) channels, purinergic receptors (P2X/P2Y), and mechanosensitive PIEZO (PIEZO 1 and PIEZO 2) channels, in sensory transmission and pain modulation. Their dysregulation, induced by chronic hyperglycemia and oxidative stress, promotes ectopic firing, altered calcium homeostasis, and glial activation, sustaining nociceptive hypersensitivity. The review further evaluates current and emerging ion channel-targeted therapeutic approaches, highlighting mechanistic insights, translational challenges, and future research directions. Recent research highlights multi-target and combination strategies, such as Naᵥ1.8 inhibition with KCNQ activation or concurrent blockade of TRPV1 and P2X3, as promising avenues offering synergistic analgesic benefits and disease-modifying potential. Advances in nanocarrier-based delivery, gene modulation, and patient-specific electrophysiological profiling further enhance translational prospects. Ultimately, the therapeutic landscape of PDN is shifting from single-channel blockade toward integrated approaches that modulate excitability, inflammation, and metabolic stress concurrently. Ion channels thus represent not only crucial mediators of PDN pathophysiology but also versatile therapeutic targets whose selective and combinatorial modulation may transform the management of diabetic neuropathic pain.

See also the graphical abstract(Fig. 1).

## Introduction

Diabetic neuropathy (DN) is one of the most prevalent chronic complications of both type 1 and type 2 diabetes, affecting nearly half of diabetic patients over the disease course and significantly diminishing quality of life, increasing the risk of foot ulceration and amputation (Pop-Busui et al., 2017[191], Gupta et al., 2025[103]). Among its clinical manifestations, painful DN is particularly distressing due to its complex pathophysiology, characterised by spontaneous burning pain, hyperalgesia, and allodynia, which remain poorly controlled by current therapies (Gupta et al., 2025[101], Yang et al., 2025[259]). Despite optimal glycaemic control and the use of pain-relieving agents such as duloxetine, pregabalin, gabapentin, or tapentadol, many patients experience suboptimal relief often accompanied with adverse effects. Thus, a shift is needed from symptomatic relief toward novel mechanism-based therapeutic strategies that address the causal drivers of neuropathic pain (Dubský et al., 2026[61], Gupta et al., 2025[101]). Over the past two decades, growing evidence has highlighted ion channel dysfunction as a key contributor to DN pathophysiology. Ion channels are integral membrane proteins that regulate the flow of ions across neuronal membranes, thereby governing neuronal excitability, action potential propagation, neurotransmitter release, and nociceptive signaling (Finnerup et al., 2021[76], Joksimovic et al., 2022[128]). 

Sensory neurons of the dorsal root ganglia (DRG) express a collection of ion channels, including voltage-gated sodium (Naᵥ), calcium (Caᵥ), and potassium (Kᵥ, KCNQ, Kir) channels, as well as transient receptor potential (TRP), purinergic (P2X, P2Y), and mechanosensitive PIEZO (PIEZO 1 and PIEZO 2) channels, that finely monitor nociceptive signaling (Alles and Smith, 2021[4], Joksimovic et al., 2022[128], Garcia-Mesa et al., 2023[84], Ślęczkowska et al., 2023[216] ). In diabetic conditions, the expression, gating kinetics, trafficking and modulation of these channels is altered by metabolic, vascular and inflammatory insults, thereby converting a metabolic neuropathy into a hyper-excitable pain generator” (Joksimovic et al., 2022[128], Asiri and Zaheen Hassan, 2023[12], Ślęczkowska et al., 2023[216]). Under hyperglycemic conditions, these channels undergo maladaptive molecular and electrophysiological remodeling due to metabolic, oxidative, and inflammatory insults, leading to hyperexcitability and spontaneous ectopic discharges in peripheral nociceptors (Feldman et al., 2017[71], Wang et al., 2024[243]). Thus, PDN may be viewed not merely as a degenerative neuropathy, but as a channelopathy-driven pain disorder, where altered ion-channel expression and function perpetuate abnormal pain transmission.

**Figure 2:**
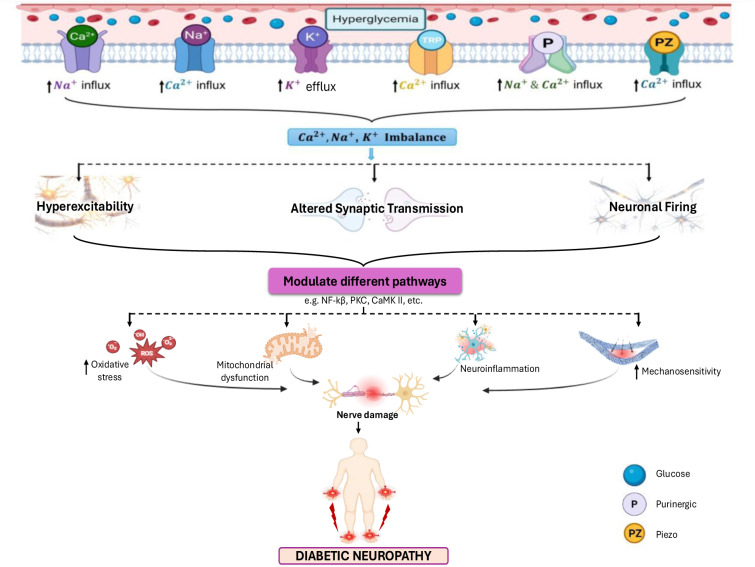
Ion channel-driven mechanisms underlying diabetic neuropathy. Chronic hyperglycemia induces dysfunction of multiple ion channel families, including voltage-gated sodium (Naᵥ), voltage-gated calcium (T-type and N-type VGCCs), potassium (Kᵥ/KCNQ), transient receptor potential (TRP), purinergic (P2X/P2Y), and mechanosensitive PIEZO channels. Aberrant activity of these channels results in a common ionic imbalance characterized by increased intracellular Na⁺ and Ca²⁺ levels and reduced K⁺ conductance. This ionic disequilibrium causes hyperexcitability, alters synaptic transmission, neuronal firing, and further activates calcium-dependent signaling cascades, particularly Ca²⁺/calmodulin-dependent protein kinase II (CaMKII), and modulates other pathways like MAPK, PKC, NF-κB, etc., which thereby lead to enhanced oxidative stress, mitochondrial dysfunction, neuroinflammation, and increased mechanosensitivity. The convergence of these pathogenic processes promotes neuronal injury, ultimately resulting in nerve damage and the development of diabetic neuropathy.

The pathogenesis of DN begins with hyperglycemia-driven metabolic stress that includes activation of the polyol pathway, accumulation of advanced glycation end-products (AGEs), mitochondrial dysfunction, oxidative stress, microvascular injury and ischemia to peripheral nerves (Callaghan et al., 2012[33], Chalotra et al., 2024[40]). These insults lead to structural damage including small-fibre degeneration, demyelination, axonal loss and functional abnormalities such as impaired axonal transport and neurotrophic support (Gupta et al., 2025[100]). However, structural nerve damage alone does not fully explain the emergence of neuropathic pain, given that many individuals with diabetic neuropathy remain pain-free and, conversely, pain may persist despite improved glycaemic control (Ang et al., 2014[11], Finnerup et al., 2021[76]). It is the maladaptive plasticity of sensory neurons and their ion channels that differentiates painful from painless neuropathy (Jayathilake et al., 2025[125]). In such cases, Na_v_ channels (particularly Naᵥ1.7, Naᵥ1.8, Naᵥ1.9) are up-regulated, increasing inward sodium currents, lowering activation thresholds, and favouring ectopic spontaneous firing (Bagal et al., 2015[15], Bigsby et al., 2022[23]). Voltage-gated calcium channels (especially Caᵥ3.2, a T-type channel, and Caᵥ2.2, an N-type channel) further contribute to after-depolarisation, calcium influx and neurotransmitter release, amplifying central nociceptive transmission. Meanwhile, potassium channels (Kᵥ1.2, Kᵥ2.2, KCNQ/M-channels, inward-rectifier Kir channels) are down-regulated or dysfunctional, thus reducing repolarisation capacity and prolonging neuronal depolarisation (Zemel et al., 2018[268], Hoffmann et al., 2021[113], Felix et al., 2025[72]). TRP channels (for example, TRPV1, TRPA1, TRPM8) integrate thermal, chemical, and oxidative stimuli, and are sensitised in DN, leading to thermal hyperalgesia and mechanical allodynia (González-Ramírez et al., 2017[92], Zhang et al., 2023[273] ). Purinergic channels (P2X3, P2X4, P2X7, and P2Y receptors) respond to ATP released from stressed or damaged tissues, promoting nociceptive signaling and neuroinflammation in peripheral and spinal circuits (Zou et al., 2023[285]). More recently, mechanosensitive PIEZO 1 and PIEZO 2 channels have been implicated in conversion of mechanical stimuli into electrical signals in sensory neurons, linking tactile allodynia in diabetes to altered mechanotransduction (Garcia-Mesa et al., 2023[84]). These multiple channel families, therefore, represent distinct yet convergent nodes of dysfunctional excitability in DN (Figure 2(Fig. 2)). 

From a therapeutic perspective, targeting these ion channels offers a mechanistically grounded alternative to empirical symptomatic treatment. Naᵥ channel blockers (targeting Naᵥ1.7/1.8) and T-type or N-type VGCC antagonists have shown analgesic efficacy in preclinical DN models, although clinical translation remains modest (Bigsby et al., 2022[23], Choudhary et al., 2023[45]). Kᵥ channel activators (e.g., KCNQ enhancers) aim to restore repolarisation capacity and reduce neuronal hyperexcitability (Yu et al., 2018[263]). TRP channel antagonists (e.g., TRPV1/TRPA1 blockers) promise to interfere with peripheral sensitisation to thermal or chemical triggers in DN (Moran et al., 2011[178], Wang et al., 2023[240]). Purinergic P2X receptor antagonists are emerging as modulators of neuropathic and inflammatory pain, and mechanosensitive PIEZO channel inhibitors present entirely novel avenues for therapeutic intervention, especially in tactile allodynia in diabetes (Gum et al., 2012[97], Garcia-Mesa et al., 2023[84], Xu et al., 2024[255]). Accordingly, DN is not a consequence of a single dysfunctional ion channel but rather the result of a network of dysregulated channels interacting within sensory, spinal, and glial circuits. Thus, a multi-target or combination therapy approach may be more fruitful, for instance, one that modulates several ion channel types or combines channel modulation with anti-oxidative, anti-inflammatory or neurotrophic strategies. However, challenges remain regarding channel selectivity, blood-brain barrier penetration, off-target toxicity, and inter-individual variability in response. Moreover, emerging technologies, including high-resolution cryo-electron microscopy (cryo-EM) for ion-channel structural elucidation, *in-silico* ligand screening, and targeted gene-silencing (siRNA, antisense oligonucleotides), have accelerated the discovery of selective channel modulators (Merino and Raunser, 2017[176], Zhu et al., 2022[284], Akhtar et al., 2025[3]). By reframing DN as an ion-channel network disorder rather than only structural nerve damage, we open a path toward mechanism-based, disease-modifying therapeutic strategies rather than mere symptom control. This review will therefore elaborate on each major ion-channel family and its role in DN, the molecular mechanisms linking metabolic injury to ion-channel remodelling, current ion-channel-targeted therapies, and emerging multi-target and combination approaches.

## Ion Channel Families and Their Roles in DN

### Voltage-gated sodium channel (VGSCs)

In 1952, Hodgkin and Huxley discovered that the inward flow of sodium ions through voltage-gated channels is essential for action potential initiation and propagation in neurons. Their experiments on the squid axon showed that these channels respond to changes in membrane voltage, permitting fast, accurate movement of Na⁺ across the membrane. Later, studies revealed that these voltage-gated sodium channels are specialized protein structures embedded in the neuronal membrane, enabling the rapid electrical signaling needed for excitability (Hodgkin and Huxley, 1952[111][110][112]). Functionally, VGSCs are essential for maintaining the excitability and conductivity of neurons, as their activation initiates the characteristic depolarizing phase of the action potential (Catterall, 2000[37]). Each VGSC is a heteromeric protein complex made up of a large pore-forming α-subunit (around 260 kDa) and another small auxiliary β-subunits (33 to 45 kDa), such as β₁, β₁A, β₂, and β₃ (Catterall, 2000[37], Catterall et al., 2005[39]). These subunits interact cooperatively to regulate the channel kinetics, expression, ensuring precise regulate neuronal signaling (Waxman, 2011[247], 2013[248]). The α-subunit gene family consists of ten unique members, named Na_v_1.1 to Naᵥ1.9, serve as voltage-gated channels. Na_x_ is a related isoform that is not voltage-gated and is involved in salt level sensing (Goldin et al., 2000[90]). The central and peripheral nervous systems express many VGSC isoforms, each of which has distinct expression patterns and biophysical properties that support certain neuronal activities (Novakovic et al., 2001[183]). Recent study has highlighted the role of α-subunit isoforms in neuropathic pain disorders and further emphasized their function in both normal and pathological conditions. Among them, painful diabetic neuropathy (DN) has drawn a lot of interest because to the aberrant expression and altered activity of specific VGSC subtypes in injured sensory neurons. These changes increase the excitability of neurons, leading to the continuous pain sensations commonly seen in DN (Wang et al., 2024[243]).

The VGSC functions in three primary states: resting, active (open), and inactivated (closed). The channel is blocked under hyperpolarized membrane potential in the resting state, which stops sodium ions from moving across the membrane. Voltage changes cause the channel to open when the membrane depolarizes, allowing Na+ ions to move quickly through the pore and start more depolarization. The intracellular loop that connects domains D3 and D4, which functions as a molecular gate to block the inner pore and stop sodium influx, mediates the channel's quick transition from activation to an inactivated state in a matter of milliseconds (Wang et al., 2017[242], Tonggu et al., 2024[228]). Naᵥ1.3, Naᵥ1.7, Naᵥ1.8, and Naᵥ1.9 are among the ten known VGSC α-subunit isoforms that are particularly relevant to the pathophysiology of neuropathic pain. These isoforms are mostly expressed in the dorsal root and trigeminal ganglia peripheral sensory neurons, where they precisely control the start and propagation of nociceptive signals (Cummins et al., 2007[50], Dib-Hajj et al., 2010[54]).

#### Naᵥ1.3 

Among the various VGSC isoforms, Naᵥ1.3 (encoded by SCN3A) has gained attention due to its dynamic expression pattern and strong association with neuronal hyperexcitability following nerve injury (Smith et al., 2018[218], Liao et al., 2023[154]). Under normal physiological conditions, Naᵥ1.3 is mostly generated in the central nervous system (CNS), where it is crucial for controlling the excitability and maturation of early neurons. On the other hand, its expression is either very inadequate or absent in the adult nervous system, indicating that Naᵥ1.3 is more involved in neuronal development than in mature sensory signaling (Cummins et al., 2001[49], Liao et al., 2023[154]). According to several studies, chronic hyperglycemia and diabetes cause aberrant upregulation (re-expression) of Naᵥ1.3 in DRG neurons. It is re-expressed in adult sensory neurons after nerve damage or diabetic neuropathy. This upregulation is considered a major contributor to neuronal hyperexcitability, a fundamental mechanism underlying the abnormal pain sensations observed in neuropathic conditions (Dib-Hajj et al., 1998[52], Black et al., 2004[25], Fukuoka et al., 2008[80]). Functionally, Naᵥ1.3 channels are characterized by rapid recovery from inactivation and a low activation threshold, enabling neurons to fire at higher frequencies and sustain repetitive discharges. These properties make Naᵥ1.3 particularly effective in driving ectopic or spontaneous firing in injured sensory neurons, one of the defining electrophysiological features of DN (Waxman et al., 1994[249], Liu et al., 2000[161], Zhao et al., 2006[280]). Multiple molecular pathways regulate overexpression of Naᵥ1.3, for example, it has been demonstrated that downregulating microRNA-30b increases Naᵥ1.3 expression in damaged DRG neurons, whereas increasing miR-30b level decreases aberrant pain responses (Su et al., 2017[220]). Additionally, intracellular modulators such as SIRT1 downregulation in the spinal dorsal horn increase acetylation of the SCN3A, further upregulating Naᵥ1.3 channel expression, enhancing neuronal excitability, and contributing to pain signaling, while Fibroblast Growth Factor 14 (FGF14) interacts with Naᵥ1.3 channels and regulates channel inactivation kinetics and influences the neuronal firing patterns and overall excitability (Martinez-Espinosa et al., 2021[171], Wang et al., 2024[245]). Additionally, the tetrodotoxin-sensitive current in damaged DRG neurons and biophysical properties of Naᵥ1.3 were similar, indicating a function for Naᵥ1.3 in injury-induced neuronal hyperexcitability. The DRG and sciatic nerves of diabetic neuropathic rats exhibit suppression of miR-214-3p, a conserved miRNA that targets Naᵥ1.3. By regulating Naᵥ1.3 in the DRG, overexpression of miR-214-3p reduces streptozotocin (STZ)-induced neuropathy, nerve conduction retardation, neural lesions, inflammation, and apoptosis (Wang et al., 2024[243]). These evidences indicate that Naᵥ1.3 upregulation under diabetic conditions contributes to aberrant neuronal firing and lowered pain thresholds, thereby intensifying the persistent burning and tingling sensations characteristic of PDN.

#### Naᵥ1.7

The VGSC Naᵥ1.7, regulated by SCN9A gene, has been identified as a crucial mediator of peripheral pain perception due to it is widely distribution in sympathetic neurons and dorsal root ganglia, where it controls neuronal firing properties. (Bang et al., 2018[16], Dormer et al., 2023[58]). By increasing small, gradual depolarizations that result in what scientists refer to as ramp currents, Naᵥ1.7 regulates the firing threshold of sensory neurons in normal physiology. This amplification prevents undesired spontaneous firing while ensuring that neurons react correctly to harmful stimuli. The channel maintains its availability at resting membrane potential, where it may react to slight depolarizations, due to its slow inactivation kinetics. However, at higher depolarized voltages, the spontaneous activation channel becomes inactive (Goodwin et al., 2022[93], Deng et al., 2023[51]). It is distinctive biophysical characteristics, such as quick activation and delayed kinetics of closed-state inactivation, that allow for precise modulation of nociceptive signaling without triggering undesired neuronal firing (Shields et al., 2012[210], Fouillet et al., 2017[77], Dong et al., 2025[57]). Peripheral nerve injury, diabetic neuropathy, and other diseases are caused by significant changes in Naᵥ1.7 expression level and functional features during pathological situations such as diabetes and chronic hyperglycemia (Hameed, 2019[105]). While Naᵥ1.7 amplifies external stimuli and depolarizes membrane potentials closer to the threshold for Naᵥ1.8 activation, gain-of-function mutations in Naᵥ1.7 may cause diabetes-induced increased sensitivity of DRG neurons (Yang et al., 2016[258]). These gain-of-function mutations may be linked to the severity of pain and may cause axonal degeneration through energy stress. For example, sensory axons with the Naᵥ1.7 G856D mutation associated with small fiber neuropathy have lower ATP level and more reactive oxygen species (Rolyan et al., 2016[203]). It has been consistently observed that DRG neurons with increased Naᵥ1.7 expression exhibit lower action-potential firing thresholds, increased sodium influx, and aberrant spontaneous neuronal activity, which cause hyperalgesia and allodynia (Li et al., 2019[152]). From a physiological perspective, Naᵥ1.7 channel play a key role in the neuronal hyperexcitability that characterizes neuropathic pain states because they sustain recurrent firing and amplify weak stimuli (Chang et al., 2018[41], Mehboob et al., 2021[175]).

#### Naᵥ1.8

Naᵥ1.8 (SCN10A) is a tetrodotoxin-resistant sodium channel that plays a key role in pain signaling. It is found mainly in pain-sensing nerve cells of the dorsal root ganglia, where it generates most of the sodium current needed to trigger action potential. Its unique properties allow pain neurons to fire repeatedly at high frequencies, which is essential for transmitting pain signals to the central nervous system (Heinle et al., 2024[107]). Under physiological conditions, Naᵥ1.8 has unique electrical properties that make it work differently from other sodium channels. Unlike typical sodium channels, it activates and inactivates at higher voltages, and shuts down slowly. More importantly, Naᵥ1.8 does not fully stop working when the neuron is depolarized, it keep some channels open and allows current to flow through. Means Naᵥ1.8 keeps working when other sodium channels have stopped, allowing pain neurons to keep firing even during sustained stimulation (Xiao et al., 2019[254]). Naᵥ1.8 channel expression and location change with nerve damage or metabolic stress. While adjacent undamaged sensory axons increase Naᵥ1.8 channel density, especially in the periphery of the nerve, Naᵥ1.8 mRNA and protein levels decrease in directly injured dorsal root ganglion neurons. The development and duration of neuropathic pain are caused by this redistribution, which encourages spontaneous firing and ectopic electrical activity (Ma et al., 2019[165]). Studies on methylglyoxal has shown that Naᵥ1.8 plays a crucial role in DN, where hyperglycemia increases the production of methylglyoxal. By activating Naᵥ1.8 expressed on the primary afferent sensory neurons, endogenously elevated methylglyoxal levels may trigger PDN (Bierhaus et al., 2012[22]). Additionally, a continuously active version of Naᵥ1.8 is produced when methylglyoxal binds to arginine residues. Thus, methylglyoxal depolarizes sensory neurons and induces posttranslational modifications in Naᵥ1.8, which appears to be the cause of primary hyperalgesia in diabetic people and animals (Bierhaus et al., 2012[22]).

In DN, reactive carbonyl species can modify channel residues and shift gating toward greater availability, amplifying nociceptive signaling even without overt axonal loss (Bierhaus et al., 2012[22]). Gain-of-function SCN10A variants found in small-fiber which delay inactivation and enhance persistent sodium influx, leading to sustained firing in DRG neurons and increased sensory excitability. These changes are consistent with the clinical pain phenotypes observed in affected patients (Garrison et al., 2014[85]). Studies have demonstrated that human pain-related tissues frequently exhibit elevated Naᵥ1.8 immunoreactivity, which is consistent with the burning and tingling sensations typical of neuropathic conditions (Bird et al., 2013[24]). The threshold for action potential generation is lowered by slight increases in Naᵥ1.8 conductance or a hyperpolarization in its activation curve, according to computational modeling, which also replicates important electrophysiological characteristics of neuropathic pain, such as aberrant action potential shape, increased repetitive firing, and elevated neuronal excitability (Kan et al., 2024[133]).

#### Naᵥ1.9

Naᵥ1.9 represents a tetrodotoxin-resistant voltage-gated sodium channel encoded by SCN11A that demonstrates preferential expression within small-diameter nociceptive neurons throughout dorsal root ganglia and trigeminal ganglia regions (Vanoye et al., 2013[234], Dib-Hajj et al., 2015[53]). This channel differs from Naᵥ1.7 and Naᵥ1.8 in that it shows activation characteristics close to resting membrane potential values, about −70 mV, allowing for the generation of low-threshold, sustained sodium currents that prolong activity during slight depolarizing events (Sleeper et al., 2000[217]). Functionally, Naᵥ1.9 operates as a subthreshold amplification mechanism through its persistent current, enhancing neuronal responsiveness to diminished or gradual stimulation patterns (Zhao et al., 2023[278]). Under normal conditions, the channel facilitates the firing of action potentials by increasing the resting membrane potential toward positive values and maintains the elevated sensitivity of the neurons, allowing them to recognize signals of inflammatory pain. To transmit strong, persistent pain signals to the brain, the channel produces prolonged, repeated firing and threshold activity (Bennett et al., 2019[20]). While Naᵥ1.9 generates complex firing patterns in healthy DRG neurons, such as evoked firing and spontaneous bursts, its potential to contribute to neuropathic pain caused by nerve damage is yet unclear. In contrast, Naᵥ1.9 is known to be associated with inflammatory pain, where inflammatory mediators either upregulate it or enhance its post-translationally to increase nociceptor response (Huang et al., 2014[117]). During diabetes, sustained hyperglycemia and oxidative stress trigger several mechanisms to raise sodium channel activity: (1) increased expression of Naᵥ1.9 in DRG neurons; (2) post-translational phosphorylation of Naᵥ1.9 via PKA/PKC pathways; and (3) ROS-dependent cholesterol oxidation, which hyperpolarizes Naᵥ1.9 activation thresholds. Notably, Naᵥ1.9 is upregulated mostly in large diameter neurons, which is an ectopic expression pattern different from its typical small fiber location and may be a factor in allodynia (Wang et al., 2024[243], Yang et al., 2025[259], Bigsby et al., 2022[23]). Following peripheral nerve injury, animal model investigations consistently demonstrate reductions in Naᵥ1.9 expression in sensory neurons (Amaya et al., 2006[7]). Various studies have described that Naᵥ1.9 knockout and antisense knockdown rodents consistently reduce inflammatory pain but have little effect on neuropathic pain, after nerve injury or baseline acute pain thresholds. This selectivity reflects differential channel regulation of inflammatory mediators such as PGE2, bradykinin, and IL-1β, which strongly potentiate Naᵥ1.9 through PKA/PKC pathways, whereas nerve injury triggers upregulation of Naᵥ1.7 and Naᵥ1.3 instead (Maingret et al., 2008[167], Kakimura et al., 2010[131], Lolignier et al., 2011[162]). Thus, Naᵥ1.9 acts as a crucial regulatory factor among voltage-gated sodium channel families, managing nociceptive neuron excitability and reactivity to inflammatory and metabolic signaling cascades, making it a significant contributor of DN (Amsalem et al., 2018[8]).

### Voltage-gated Calcium channel

Voltage-gated calcium channels are complex cell membrane proteins that, upon depolarization, allow calcium to enter the cell in response to changes in voltage (Dolphin, 2016[56]). This calcium influx into neurons is essential for translating electrical activity into physiological responses, such as neurotransmitter release, modulation of neuronal excitability, regulation of gene expression via calcium-responsive transcription factors, and activation of multiple intracellular signaling cascades that mediate synaptic plasticity and neuronal adaptation under both physiological and pathological conditions (Zamponi et al., 2009[267], Hering et al., 2018[109]). VGCCs are multi-subunit protein complexes composed of a pore-forming α1 subunit that conducts Ca2+ ions, together with regulatory auxiliary subunits including α2δ, β, and γ. The α2δ subunit enhances current density and modulates gating properties, the β subunit is essential for membrane trafficking of the channel complex and also regulates channel gating kinetics and voltage-dependent activation, whereas, the γ subunit, found in few VGCC complexes, negatively regulates channel current and gating kinetics rather than trafficking (Dolphin, 2016[56]). The former α_1_ subunit, determines the distinct biophysical and pharmacological properties of L-(Caᵥ1), N-(Caᵥ2.2), P/Q-(Caᵥ2.1 ), R-(Caᵥ2.3), and T-(Caᵥ3) type channels (Zamponi, 2016[266]). These calcium channels open during neuronal signaling when action potentials reach presynaptic terminals, where calcium influx rises sharply and Ca^2+^ ions bind to synaptotagmin, a calcium-sensing protein on synaptic vesicles, triggering the assembly and activation of Soluble N-ethylmaleimide-sensitive factor attachment protein receptors (SNARE) protein complexes that mediate vesicle fusion with the presynaptic membrane. Neurotransmitters are released into the synaptic cleft, where they bind postsynaptic receptors to transmit signals between neurons (Young and Neher, 2009[261]). In addition to this rapid signaling, Ca^2+^ ions can trigger longer term alterations in neuronal excitability and gene expression that are responsible for pain sensitization and chronic pain disorders (Hagenston and Simonetti, 2014[104]). In sensory neurons and nociceptors specifically, calcium channel regulation directly determines hyperexcitability of neurons, which is a key feature of neuropathic pain (Altier and Zamponi, 2004[6]).

Sustained hyperglycemia in DN disrupts normal calcium homeostasis in sensory neurons by activating many metabolic pathways that produce oxidative stress and reactive oxygen species. leads to the upregulation of calcium channel proteins in the DRG, particularly the auxiliary α2δ-1 subunit, which enhances calcium influx and drives the neuronal hyperexcitability underlying diabetic pain (Luo et al., 2001[164], Fernyhough and Calcutt, 2010[74]). Recent studies reveal the association between Ca^2+^ activity and neuroinflammation, as well as interactions between neurons and glial cells. When sensory neuron calcium signaling is disrupted, glutamate and substance P are released, thereby increasing astrocyte and microglia level in the spinal cord, which causes inflammation and sustains the hyperactivity of neurons (Zamponi et al., 2009[267]). Therefore, calcium channels are not only responsible for initiating aberrant neuron firing but also for causing cellular stress, inflammation, and nerve damage that sustain DN discomfort. 

#### N-Type Calcium Channel (Caᵥ2.2) 

N-type calcium channel (Caᵥ2.2) an important voltage-activated calcium channel subtype that control presynaptic neurotransmitter release in nociceptive pathways and is encoded by CACNA1B gene (Pearson, 2007[188]). These channels, abundantly expressed in DRG neurons and their central terminals in the spinal dorsal horn, act as molecular transducers that couple membrane depolarization to calcium influx, causing release of excitatory neurotransmitters such as glutamate, substance P, and calcitonin gene-related peptide (CGRP) from synaptic vesicles (Chi et al., 2009[44], Park and Luo, 2010[187], Catterall, 2011[38]). Under physiological conditions, these regulate calcium-dependent processes and maintain normal sensory signal transmission. In DN conditions, this homeostatic process is severely disrupted by chronic hyperglycemia and related metabolic abnormalities, altering Caᵥ2.2 from a physiological mediator into a pathological cause of neuronal damage and chronic neuropathic pain (Joksimovic et al., 2022[128]).

Persistently elevated blood glucose leads to the accumulation of ROS and AGEs in the body, which triggers MAPKs, involved in downstream signaling pathways (González et al., 2023[91]). In Metabolic dysfunction, PKC, calmodulin-dependent protein kinase II (CaMKII), and MAPK kinases directly phosphorylate Caᵥ2.2 channels, increasing their opening frequency and accelerating their activation kinetics, allowing upsurged calcium influx through existing channel proteins, while concurrent upregulation of CACNA1B transcription via CREB and NF-κB increases channel expression, producing a synergistic amplification of presynaptic calcium influx (Szymanowicz et al., 2024[221]). Chronic hyperglycemia also causes the α2δ-1 auxiliary subunit (CACNA2D1) to be upregulated, which increases Caᵥ2.2 channel trafficking to the presynaptic membrane and, thereby, enhances calcium influx during action potentials (Bauer et al., 2009[19]). In nerve terminals, excessive influx dysregulates Caᵥ2.2 channels, triggering mitochondrial calcium overload, reducing ATP production, and initiating the axonal degeneration typical of progressive DN.

#### L-Type Calcium Channels (Caᵥ1.2 and Caᵥ1.3)

The L-type channels (Caᵥ1.2 and Caᵥ1.3) located in neuronal soma and dendrites are involved in signal transduction and provide a molecular intermediate between calcium influx and intracellular calcium signaling pathways rather than vesicle release (Zhang et al., 2006[271]). In normal conditions, calcium entry via these channels results in activation of calcineurin, PKA and CaMKII, which phosphorylate the downstream transcription factors cAMP response element-binding protein (CREB) and Nuclear Factor of Activated T cells (NFAT). Gene regulation by activity-dependent calcium signaling is essential for maintaining neuronal plasticity and metabolic balance (MacDonnell et al., 2009[166], Lee and Fields, 2021[149]). However, in DN, chronic hyperglycemia and oxidative stress transform this adaptive calcium signaling into a pathological process.

ROS produced from hyperglycemia activate various PKC isoforms (PKC-β and PKC-ε) (Yang et al., 2009[257]), which can phosphorylate Caᵥ1.2 channels in some contexts, but in acute or direct glucose-mediated hyperglycaemic signaling, the primary kinase phosphorylating Caᵥ1.2 at Ser1928 is PKA. Hyperglycemia also activates PKA-mediated phosphorylation at the Ser1928 site on Caᵥ1.2, enhancing Caᵥ1.2 channel activity and elevated calcium entry into neurons under hyperglycemic conditions (Nystoriak et al., 2017[184]). The resultant increase in presynaptic and somatic calcium influx exceeds physiological values. The excessive L-type calcium load also surpasses the mitochondrial calcium retention threshold, inhibiting oxidative phosphorylation, reducing neuronal ATP production and inducing opening of the mPTP (Strubbe-Rivera et al., 2021[219], Ivanova et al., 2025[121]). Diminished caveolin-1 expression promotes demyelination through enhanced Erb B2 signaling in Schwann cells. Loss of Cav-1 impairs myelin maintenance, causing secondary axonal damage and reduced neuroprotection (McGuire et al., 2009[173]). Increased calcium also activates the CaMKII and ERK/MAPK cascades, which reduce anti-apoptotic gene expression (Bcl-2) while promoting pro-apoptotic signaling. This transcriptional alteration drives the apoptosis of sensory neurons, accounting for the progressive loss of nerve fibers in DN (Timmins et al., 2009[226], Chung et al., 2018[46]).

#### T-Type Calcium Channels (Caᵥ13.2)

T-type calcium channels, particularly (Caᵥ3.2), encoded by the CACNA1H gene (Cain et al., 2018[31]), key regulators which control subthreshold excitability and burst firing in nociceptive pathways DRG neurons. especially C-fiber nociceptors, express Caᵥ3.2 in abundance at the soma and peripheral terminals. These low-voltage-activated channels open near resting membrane potentials act as molecular amplifiers, converting small membrane depolarizations into calcium influx, thereby promoting repetitive firing and improving nociceptive signaling in the spinal dorsal horn (Todorovic and Jevtovic-Todorovic, 2011[227], Voisin et al., 2016[238]). This low-threshold calcium influx stabilizes neuronal firing rates and preserves normal sensory response under physiological conditions. Relying on CACNA1H transcriptional upregulation, posttranslational changes, mainly N-linked glycosylation, are the main cause of the approximately two-fold increase in Caᵥ3.2 activity in diabetic DRG neurons , T-type calcium currents are amplified, the Caᵥ3.2 pore-forming subunit is phosphorylated, oxidative changes (glycation and S-nitrosylation) occur, and neuronal excitability exceeds physiological thresholds (Joksimovic et al., 2020[127]). Elevated low-threshold calcium influx disrupts intracellular homeostasis, facilitates mitochondrial calcium overload above the organelle retention capacity, and impairs oxidative phosphorylation, ultimately decreasing ATP production and contributing to progressive axonal degeneration characteristic of diabetic neuropathy (Gleichmann and Mattson, 2011[89]). Additionally, persistent Caᵥ3.2 hyperactivity strengthens both peripheral and central sensitization mechanisms in nociceptive pathways. Increased glutamate and neuropeptide release in the dorsal horn, ectopic spontaneous firing at the peripheral terminal, and long-term potentiation-like plasticity are all facilitated by enhanced T-type channel activity, which sustains chronic diabetic neuropathic pain (Jacus et al., 2012[123]).

### Voltage-gated potassium channels

Voltage-gated potassium channels (VGKC) are the largest superfamily of voltage-gated ions channels encompassing approximately 80 genes distributed into 12 subfamilies named as (Kᵥ1 to Kᵥ12) (Zheng and Chen, 2024[282]), playing key roles in several aspects of regulating neuronal excitability, facilitating K+ swift and selective movement across the cell membrane, controlling firing frequency, generating action potential repolarization, and stabilizing resting membrane potential in sensory neurons. In DRG neurons, VGKC acts as an electrical brake, counterbalancing depolarizing sodium currents and ensuring that neurons do not fire excessively under normal physiological conditions. Structurally, VGKC characteristically consists of tetrameric α-helices (S1-S6) subunits, in which channels S1-S4 subunits contribute to the pore-forming domain of the channels (Grizel et al., 2014[96]). VGKC is categorized into different subfamilies in the DN context, mainly involving Kᵥ1, Kᵥ3, Kᵥ4, and Kᵥ7.

Under normal conditions, Kᵥ regulate accordingly by repolarizing the membrane after each action potential and limiting repetitive firing. However, hyperglycemia and oxidative stress lead to altered normal function and post-translational regulation of the Kᵥ subtypes. Reduction in Kᵥ_1_ and Kᵥ_4_ channel availability have been observed in rodent diabetic models. Some inflammatory mediators, such as TNF-α, IL-1β, and prostaglandins, further suppress Kᵥ function via PKC and ERK-dependent phosphorylation pathways, diminishing K⁺ outflow and amplifying nociceptive firing, producing neuronal hyperexcitability, which causes DN (Velázquez et al., 2007[236]). Numerous studies demonstrate that Kᵥ channels are among the most consistently downregulated ion channels in injured or metabolically stressed DRG neurons (Zheng and Chen, 2024[282]). 

#### Kᵥ1

Among the various subtypes of VGKC, especially Kᵥ_1.1_, Kᵥ_1.2_, and Kᵥ_1.4_, encoded by KCNA1, KCNA2, and KCNA4, are highly expressed in DRG neurons, with the expression of Kᵥ_1.1 _and Kᵥ_1.2 _predominantly in large-diameter neurons, and the subunit Kᵥ_1.4_ in small-diameter neurons. These are present on the soma and juxtaparanodal regions of myelinated axons. These channels mediate low threshold, slowly inactivating outward K⁺ currents that stabilize the resting membrane potential and shorten the action potential, thereby limiting repetitive firing and ectopic discharges (Rasband et al., 2001[196]). These channels act as an electrical brake, they stop repolarization, ultimately increasing depolarization in neurons and more likely to fire spontaneously. 

In a streptozotocin (STZ) rat model of painful diabetic neuropathy, Cao et al. (2010[35]) showed that in hyperglycemia BDNF expression is increased in DRG neuron via tyrosine phosphorylation, causing a reduction in Kᵥ currents, especially in Type-A current (Cao et al., 2010[35]). In myelinated nerve fibers, Kᵥ_1_ channels are present at juxtaparanodal regions adjacent to nodes of Ranvier, where they play essential roles in action potential repolarization and nerve excitability. Hyperglycemia causes altered distribution and reduced presence of Kᵥ1.2 subunits in juxtaparanodal regions. This mis-localization disrupts the normal spatial organization of ion channels necessary for saltatory conduction (Zenker et al., 2012[269]). According to Zhao et al. (2017[279]), hyperglycemia-induced nerve damage enhances DNMT3a in DRG neurons, which reduces Kᵥ1.2 expression and methylates the Kcna2 promoter. This decrease in Kᵥ1_.2 _contributes to DN by lowering potassium currents and increasing neuronal excitability (Zhao et al., 2017[279]).

Kᵥ_1_ channel dysfunction is caused by metabolic and oxidative stress processes via numerous mechanisms. Hyperglycemia activates PKC, especially the β and δ isoforms, by increasing the production of diacylglycerol (DAG), while AGE accumulation in the tissue lowers Kᵥ channel current density and significantly decreases expression at both gene and protein level, initiating downstream signaling cascades that downregulate channel expression (Liu et al., 2019[160]). PKC-mediated phosphorylation inhibits Kᵥ_1_ channel activity and decreases its surface expression via inducing ubiquitination-dependent endocytosis and lysosomal degradation, among other consequences. Because PKC activation may activate NADPH oxidase 2, which produces reactive oxygen species (ROS), the PKC pathway is also associated with oxidative stress. Through both functional inhibition and decreased expression, this PKC-ROS axis contributes to decreased Kᵥ channel activity (Du et al., 2021[60]). Also pro-inflammatory cytokines, particularly IL-1β, IL-6, and TNF-α, are elevated in hyperglycemia and contribute to Kᵥ1 channel dysregulation. These cytokines promote neuroinflammation, microglial activation, and neuronal hyperexcitability. Inflammatory mediators can indirectly affect Kᵥ_1_ channel expression and function through activation of intracellular signaling cascades, including NF-κB and MAPK pathways. Reduction of Kᵥ channel expression leads to several pathophysiological effects such as membrane potential depolarization, hyperpolarized action potential threshold, and increased spontaneous firing rates of sensory neurons (Andrei et al., 2025[10]). 

#### Kᵥ3

The Kᵥ3 VGKC triggers a high-voltage-activating A-type Kᵥ current found in axons and nerve terminals. These high-voltage-activated K⁺ channels have fast activation and very fast deactivation, classically associated with fast-spiking neurons. In peripheral sensory pathways, the main Kᵥ3 subunit with clear nociceptive relevance is Kᵥ3.4, encoded by the KCNC4 gene (Zemel et al., 2018[268]). Immunohistochemical and electrophysiological studies show that Kᵥ3.4 is strongly expressed in small-diameter nociceptive DRG neurons, especially C-fibers, with channel protein located in soma, axons and peripheral terminals. Kᵥ3.4 currents in DRG neurons generate a fast-inactivating A-type outward K⁺ current that helps narrow the action potential and limit Ca²⁺ entry at nerve endings, thereby regulating nociceptive signals in a homeostatic manner (Ritter et al., 2012[200]). 

Ritter et al. (2015[201]) demonstrated that small-diameter DRG neurons from spinal cord injury animals show a marked reduction in Kᵥ3.4-mediated A-type potassium current due to altered channel trafficking and changes in the inactivation properties of the Kᵥ3.4 N-terminal domain, which normally ensures rapid current inactivation. As a result of this dysregulation, action potentials become broader and allow greater calcium influx into the terminals of nociceptive neurons (Ritter et al., 2015[201]). Additionally, in the streptozotocin (STZ) model of type 1 diabetes, Cao et al. (2010[35]) reported a substantial decrease in total voltage-gated potassium currents, with the downregulation of Kᵥ3.4 channel. This decrease in 3.4 current results in membrane depolarization and increasing hyperexcitability, consequently causing DN (Cao et al., 2010[35]).

#### Kᵥ4 

The VGKC, Kᵥ4 (Kᵥ4.1, Kᵥ4.2, Kᵥ4.3, encoded by KCND1, KCND2, and KCND3, respectively), is fundamentally different from the Kᵥ_1_ and Kᵥ_3_ families in their biophysical properties and physiological roles in nociceptors. These channels activate at subthreshold voltages (-40 to -20 mV), rapidly activate and inactivate in response, producing a transient outward K+ current uniquely characterized by a fast recovery from inactivation (Covarrubias et al., 2008[48], Zemel et al., 2018[268]). 

Chronic hyperglycemia leads to hyperexcitability in neurons. Kᵥ_4_ channels undergo pronounced dysregulation. Several studies showed that sciatic nerve injury, spinal nerve ligation, and trigeminal nerve models consistently show a selective downregulation of Kᵥ_4.2_ and Kᵥ_4.3_, leading to a marked reduction in A-type current density (Viatchenko-Karpinski et al., 2018[237]). Thereby extending action potential, increasing calcium influx, Kᵥ_4.3_ downregulation uniquely contributes to cold allodynia, and selective pharmacological obstruction of Kᵥ_4.3_ in otherwise healthy animals is sufficient to induce cold hypersensitivity, providing clear evidence that this subunit acts as a critical determinant of cold-pain processing (Kanda et al., 2021[134]). Similarly, BDNF elevations, MAPK phosphorylation via Thr602, PKC activation, and inflammatory cytokines alter the expression of Kᵥ_4_ subtypes (Carrillo-Reid et al., 2019[36]). In such conditions, Kᵥ4.2 and Kᵥ_4.3_ mRNA levels are reduced approximately 40-50 % in DRG neurons, with protein level similarly decreased, resulting in a reduction in total Kᵥ4 current density leads to DN.

#### Kᵥ7

Kᵥ_7_ channels, also known as M channels (Kᵥ_7.1_ to Kᵥ_7.5_ encoded by KCNQ1-KCNQ5), are VGKC with interesting biophysical properties (slow activation and deactivation, no inactivation, and a threshold for activation below -60 mV) (Greene and Hoshi, 2017[95]). The K^+^ current is activated near the resting membrane potential. These are expressed in small-medium DRG neurons, acting as an excitability stabilizer. Downregulation or inhibition of Kᵥ7 channels leads to enhanced excitability in central and peripheral neurons, with rapid depolarization and spontaneous firing (Barkai et al., 2017[17]). Additional injury model studies demonstrated that peripheral nerve damage increases expression of the transcriptional repressor (REST) in DRG neurons which suppresses Kcnq2 transcription and leads to a marked reduction in Kᵥ_7.2_ expression. A similar breakdown occurs in DN (Rose et al., 2011[204]), where chronic hyperglycemia reduces expression of KCNQ2, KCNQ3, and KCNQ5 in DRG neurons and decreases Kᵥ_7.5_ immunoreactivity in small nociceptive cells, leading to a pronounced reduction in M-current. As a result, diabetic DRG neurons shift to a depolarized, hyperexcitable state characterized by lowered spike threshold and spontaneous activity . Like Kᵥ_1_, Kᵥ_3_, and Kᵥ_4_ channels, MAPK phosphorylation, inflammatory cytokines, and PKC activation modulate Kᵥ_7_ channel expression (Yu et al., 2018[263]). 

### Transient Receptor Potential (TRP)

Transient Receptor Potential (TRP) channels are ion channels family members of non-selective cation that play a central role in sensory transduction, particularly in nociception, thermosensation, and mechanosensation (Zheng, 2013[281]). These ion channels were first discovered through genetic studies in the fruit fly *Drosophila*, where the TRP mutant exhibited transient electrical responses to light exposure. These channels are widely expressed in peripheral sensory neurons, especially in small-diameter dorsal root ganglion (DRG) neurons that give rise to C-fibers and Aδ-fibers. TRP channels are classified into various types, including TRPV, TRPA, and TRPM8, which are involved in the development of diabetic neuropathy. All TRP subtypes have six transmembrane domains(S1-S6) and a pore-forming loop located in the N- and C-terminal intracellular regions between S5th-S6th (Zhang et al., 2023[273]). However, TRP channels differ in that their activity is only weakly voltage dependent and is strongly regulated by ligand binding, post-translational modifications, membrane phospholipids, and protein-protein interactions (Yue et al., 2015[265]). This mode of regulation allows TRP channels to respond dynamically to ongoing cellular conditions. Activation of most TRP channels results in calcium influx that can directly affect the membrane excitability and simultaneously engage downstream signaling pathways involving kinases, phosphatases, and transcriptional regulators (Du and Liu, 2025[59]), leading to cause DN.

#### TRPV1

TRPV1, originally named vanilloid receptor 1 is a noxious heat-activated channel and commonly referred as the capsaicin receptor. It was initially described as a polymodal receptor that is activated by three pain-producing stimuli: vanilloid compounds (capsaicin, resiniferatoxin), at a temperature above approximately 43 °C and low pH <5.9 in chronic hyperglycemic conditions, metabolic stress and inflammatory mediators lower this thermal activation threshold (Du and Liu, 2025[59]). As a resultant Protein kinase A and protein kinase C-dependent phosphorylation sensitizes TRPV1, allowing it to open at physiological temperatures. As a result, normally non-painful warmth is recognized as burning pain. This mechanism explains the prominent thermal hyperalgesia and heat intolerance (Uchytilova et al., 2021[231]) observed during the early stages of DN. In pathological conditions TRVP1 decrease the expression of unmyelinated C-fibers which are primarily responsible for heat pain detection, while functional TRPV1 activity increases in medium-diameter A-fibers. This redistribution alters thermal sensitivity and contributes to abnormal temperature discrimination. Previous studies showed that impaired TRPV1 desensitization prolongs heat-evoked responses, enhancing thermal pain under repeated or sustained warm stimuli (Gao et al., 2024[81]). 

As the disease progresses, TRPV1 undergoes fiber-specific redistribution. Its expression decreases in small unmyelinated C-fibers, which are primarily responsible for heat pain detection, while functional TRPV1 activity increases in medium-diameter A-fibers. This redistribution alters thermal encoding and contributes to abnormal temperature discrimination (Kim et al., 2008[137]). Experimental models further show that impaired TRPV1 desensitization prolongs heat-evoked responses, enhancing thermal pain under repeated or sustained warm stimuli (Luo et al., 2019[163]).

#### TRPV2

TRPV2, was discovered as a structural homologue of TRPV1 with 50 % amino acid identity, which is a very high-threshold heat sensor, activated at temperatures above ~52 °C and swelling, primarily expressed in neuronal and non-neuronal cells Aδ Aβ nociceptors fibers of DRG, trigeminal ganglia(TG) (Fricke and Leffler, 2024[79]). Its activation corresponds to extreme thermal stimuli rather than physiological warmth. Under inflammatory conditions, TRPV2 function is enhanced by growth factors, particularly Insulin-like Growth Factor-I (IGF-I). Activates the PI3-kinase signaling pathway, which triggers the rapid translocation of TRPV2 channels from intracellular to the plasma membrane, thereby sensitizing neurons to noxious stimuli (Kojima and Nagasawa, 2007[143]).

#### TRPV3

TRPV3 is a warm-sensitive channel, activated in the range of approximately 30-39 °C, with increased responses to higher noxious thermal stimuli, and expressed predominantly in epidermal keratinocytes rather than in sensory neurons, including TRG and TG. TRPV3 is also strongly activated and sensitized by camphor, irritants extracted from thyme, oregano, savory, and cloves (Lei and Tominaga, 2025[151]). In normal physiology of skin, TRPV3 in basal keratinocytes contributes to harmless warmth detection and epidermal homeostasis by mediating Ca²⁺ influx and triggering the release of paracrine mediators such as ATP, prostaglandins, and nitric oxide, which regulate proper warm sensation (Mandadi et al., 2009[168], Miyamoto et al., 2011[177]). In hyperglycemic conditions caused by phospholipase C and PKC activations, keratinocytes are significantly reduced. This down-regulation of keratinocyte TRPV3 contributes less to early painful hyperalgesia and more to progressive sensory loss, including reduced warm sensation and thermal hypoesthesia, by weakening keratinocyte nerve cross-talk (Facer et al., 2007[68], Geraldes and King, 2010[86]).

#### TRPV4

TRPV4 is a polymodal, non-selective cation channel belonging to the vanilloid subfamily of TRP channels. It is activated by mild hypotonic stress, shear stress, cell swelling, and innocuous warmth, typically above ~27 °C. In addition to physical stimuli, it can be sensitized by chemical mediators such as 4α-phorbol esters, low pH, citrate, nitric oxide, endocannabinoids, and arachidonic acid metabolites (Heller and O'Neil, 2007[108], Rodrigues et al., 2022[202]). The TRPV4 channel is widely expressed, including in DRG, cutaneous A- and C-fiber terminals, keratinocytes, and vascular endothelium. This distribution supports its dual role in sensory transduction and tissue homeostasis. Genetic deletion or knockdown studies show that TRPV4 contributes to mechanosensation and osmotic sensitivity, with TRPV4-deficient animals showing increased mechanical pain thresholds and altered responses to warm temperatures (Liedtke, 2007[155], Boudaka et al., 2020[27]). 

In pathological conditions, TRPV4 plays a prominent role in inflammatory and neuropathic pain. It is strongly engaged in mechanical and osmotic hyperalgesia following exposure to inflammatory mediators such as prostaglandin E₂ and serotonin. Sensitization of TRPV4 occurs through intracellular signaling pathways involving PKA, PKC, and Src family kinases. Protease-activated receptor-2 activation during inflammation further enhances TRPV4 function, leading to increased release of neuropeptides such as substance P and calcitonin gene-related peptide (CGRP) from primary afferents and contribute to painful DN (Rodrigues et al., 2022[202]).

#### TRPVA1

TRPVA1 is a TRP subfamily member; it is a protein that is overexpressed in a liposarcoma cell line and is distinguished by the presence of many ankyrin repeat motifs on the cytosolic amino-terminal domain (TRPAnkyrin). Because of its Drosophila homologue, it functions as a sensor for mechanical stimuli and contributes to mechanical nociception (Kwan et al., 2006[144]). It is expressed in inner ear, intestine myenteric plexus neurons, motor neurons, postganglionic sympathetic neurons, lung fibroblasts, and trigeminal and DRG neurons. Physical stimuli, such as extreme cold (less than 18 °C), as well as strong substances like mustard, garlic, wintergreen, clove, ginger, and cinnamon oils, activate TRPVA (Anand et al., 2008[9]). These all cause sharp, painful burning or tingling feelings. Peripheral activation of the TRPA1 channel depolarizes the nerve ending because of the influx of sodium ions, which can cause hyperexcitability and action potentials in pain-mediating nerve fibers (Koivisto et al., 2014[140]). The TRPA1 channel may be implicated in mechanical hyperalgesia and cold allodynia, according to a number of behavioral model studies (Iannone et al., 2023[118]). Reactive substances including reactive oxygen species (ROS) and inflammatory mediators like PKC, PKA, and bradykinin, which sensitize TRPA1 via PLC-dependent pathways, are among the additional stimuli that activate the TRPA1 channel (Aubdool et al., 2016[13]). Additionally, another factor is electrophilic activation. TRPA1 reacts with an electrophilic compound like Methylglyoxal, 4-hydroxynonenal, and nitrogen species bind with TRPA1 and open the Ca²⁺ and Na⁺ influx into the nociceptor the neuron becomes depolarized and hyperexcitable, leading to Spontaneous firing (ongoing burning pain). Lower threshold to touch and cold (mechanical and cold hyperalgesia), which ultimately causes DN (Eberhardt et al., 2012[63]).

#### TRPM8

Transient Receptor Potential Melastatin 8 (TRPM8) is a non-selective, calcium-permeable cation channel, the principal detector of cold sensation. It is activated by innocuous cooling (approximately 23-28 °C) and by cooling compounds such as menthol, eucalyptol, and icilin, with voltage-dependent gating properties (Izquierdo et al., 2021[122]). First discovered in the prostate gland as an androgen-responsive channel, it is a thermally regulated channel that is activated *in-vitro* by neurons derived from both TG and DRG. This is consistent with the percentage of cultured sensory neurons responding to cold and menthol (McKemy, 2007[174]). Its activity is modulated by intracellular calcium, pH, phosphatidylinositol-4,5-bisphosphate (PIP₂), and protein kinase C signaling (Yudin and Rohacs, 2012[264]). In normal physiology, TRPM8 is upregulated, acting like a built-in natural painkiller that activates whenever you experience cool sensations. In chronic hyperglycemia and inflammatory cytokines bind to protein Gαq and inhibits TRPM8 At the same time, inflammatory signaling activates an enzyme (calcineurin) response to cell surface become internalized as a resultant TRPM8 expression in the DRG decline and lose their natural cooling-based pain relief (Proudfoot et al., 2006[193], Zhang, 2019[275]). Without TRPM8, cool sensations no longer trigger the release of endogenous painkillers.

### Purinergic receptor

In 1972, Geoffrey Burnstock proposed the concept of purinergic hypothesis, demonstrating that adenosine 5'-triphosphate (ATP) functions as a neurotransmitter in noradrenergic, noncholinergic (NANC) inhibitory nerves supplying the guinea-pig Taenia coli. which are a class of cell-surface receptors that mediate the extracellular actions of purine and pyrimidine nucleotides, primarily ATP, ADP, UTP, UDP, and nucleoside adenosine (Burnstock and Wood, 1996[30]). Unlikely, Purinergic signaling functions in both synaptic and non-synaptic contexts and plays a central role in intercellular communication during physiological stress, tissue injury, and inflammation. ATP, which is normally enclosed within cells, is released into the extracellular space during mechanical stimulation, metabolic stress, hypoxia, or cell damage, where it functions as a signaling molecule rather than an energy source (Rhett et al., 2014[198]). In chronic hyperglycemia, oxidative stress, and inflammation increase extracellular ATP release from neurons, Schwann cells, endothelial cells, and activated immune cells. This sustained ATP enhances Ca^++^ and Na+ permeability, leading to neuroinflammation, neuronal hyperexcitability, and pain sensitization (Hu et al., 2023[114]).

Purinergic receptors are broadly classified into two families: P1 receptors activated by adenosine, and P2 receptors, which respond to nucleotides/ATP. P2 receptors are further subdivided into P2X receptors, which are ligand-gated ion channels that mediate rapid ionic fluxes, and P2Y receptors, which are G protein-coupled receptors that regulate intracellular signaling pathways. Together, these receptors regulate neuronal excitability, synaptic transmission, immune cell activation, vascular tone, and glial function. P2X3, P2X7, and P2Y12 are strongly involved in DN (Burnstock, 2018[29]). 

#### P2X3

P2X3 is a ligand-gated ion channel of purinergic receptor that exists as homomeric P2X3 or heteromeric P2X2/3 complexes, which are expressed primarily in nociceptive neurons of the peripheral sensory neurons and are characteristically marked by rapid activation and rapid desensitization, especially in small to medium-diameter neurons of DRG, TG (Brederson and Jarvis, 2008[28]). During tissue stress, mechanical stimulation, or mild injury, extracellular ATP is released, which activates P2X3 receptors and induces Na⁺ and Ca²⁺ influx, leading to short‑lasting depolarization and a protective acute pain signal (Giniatullin and Nistri, 2023[88]). However, this ATP-mediated signaling is normally transient and tightly regulated. In persistent hyperglycemia, nerve injury, inflammation, mitochondrial dysfunction, and chronic metabolic stress, this transient signaling becomes dysregulated, resulting in elevated extracellular ATP levels and increased expression and sensitivity of P2X3 receptors, making sensory neurons hyperexcitable and abnormal spontaneous firing, which clinically manifests as burning pain, tingling, and sensory hypersensitivity (Xiang et al., 2008[253], Shcherbatko et al., 2016[209]). Thus, the P2X3 receptor is a central molecular mediator of ATP‑mediated peripheral pain transduction and, due to its predominant localization on peripheral sensory neurons, is considered an important therapeutic target in chronic painful conditions such as diabetic neuropathy (North, 2004[182]).

#### P2X7

The P2X7 receptor is a member of the purinergic P2X family and functions as an ATP-gated ligand-gated ion channel, which differs from other P2X receptors because it requires high extracellular ATP concentration for activation (Martínez-Cuesta et al., 2020[170]). Structurally, the P2X7 receptor is homotrimeric and has a long intracellular C-terminal tail (239 amino acids), which plays an important role in its downstream signaling. Upon short-term ATP binding, the P2X7 channel allows Na⁺ and Ca²⁺ ions to enter the cell and K⁺ to exit, but when ATP exposure becomes prolonged, the receptor forms a large non-selective pore, which markedly increases membrane permeability and disturbs cellular homeostasis (Santana et al., 2024[206]). P2X7 receptor expression occurs predominantly on non-neuronal cells such as microglia, macrophages, astrocytes, and Schwann cells, whereas its expression on sensory neurons is limited, and therefore this receptor is more involved in neuroinflammation than in direct pain initiation. In pathological conditions such as nerve injury, chronic inflammation, or metabolic stress, extracellular ATP levels persistently increase, which continuously activates P2X7 receptors (Kaczmarek-Hajek et al., 2018[129], Hu et al., 2022[115]). This activation stimulates microglial cells and the NLRP3 inflammasome, thereby activating pro-inflammatory cytokines such as IL-1β and IL-18 are released (Wang et al., 2020[241]). In diabetic neuropathy, chronic hyperglycemia induces oxidative stress, mitochondrial dysfunction, and inflammation, which further increases ATP release and P2X7 receptor activation leading to increases the excitability of dorsal horn neurons, and develops central sensitization, which clinically manifests as persistent neuropathic pain (Chen et al., 2022[43]). Thus, the P2X7 receptor is a key mediator of ATP-mediated neuroinflammatory signaling and is considered an important therapeutic target in chronic neuropathic conditions such as painful diabetic neuropathy.

#### P2Y12

P2Y12 is a Gi-protein ionotropic purinergic receptor their primary ligand is ADP and mainly found more expressed in non-neuronal cells especially satellite glial cells of the DRG and microglia of the CNS than in neurons (Kawaguchi et al., 2015[135]). Under normal physiological conditions, P2Y12 plays a limited role in glial-neuronal communication and cellular homeostasis (Sipe et al., 2016[213]), but its role becomes quite prominent in pathological states such as chronic hyperglycemic condition and nerve injury (Guo et al., 2018[99]). During hyperglycemia and nerve stress, extracellular ATP/ADP is released from neurons, which activates the P2Y12 receptor, resulting in satellite glial cell activation, evidence of which is seen as an increase in GFAP expression. The activated P2Y12 receptor stimulates the downstream p38 MAPK signaling pathway, which increases the release of pro-inflammatory cytokines such as IL-1β and TNF-α. These inflammatory mediators enhance neuronal excitability and make sensory neurons hyper-responsive (Yi et al., 2018[260]), whose clinical manifestation appears in the form of mechanical and thermal hyperalgesia. In experimental diabetic rat models, P2Y12 receptor mRNA and protein expression have been found to be significantly increased in the DRG, and after gene silencing marked reductions have been observed in p38 MAPK activation, cytokine release, glial activation, and pain behaviors (Guo et al., 2018[99]). Thus, the P2Y12 receptor is considered not a primary trigger for pain initiation but rather a central molecular mediator in the maintenance and amplification of diabetic neuropathic pain, making it an important target for therapeutic intervention (Tozaki-Saitoh et al., 2008[229]).

### PIEZO

PIEZO channels are non-selective trimeric cation mechanosensitive channels that sense and transduce membrane tension, stretch, shear stress, and osmotic changes into cellular responses through a process known as mechanotransduction (Gupta et al., 2025[102]). These trimeric cation channels possess a unique propeller-like structure with three blade-shaped subunits. other mechanosensory tissues. These intracellular activations trigger Ca²⁺ overload, oxidative stress, inflammatory signaling, and neuronal dysfunction, which leads to DN (Coste et al., 2010[47]).

#### PIEZO 1

In diabetic neuropathy, PIEZO 1, a mechanosensitive ion channel expressed in neurons, Schwann cells, endothelial cells, and immune cells, plays a crucial role at the junction between cellular signaling and mechanical stresses. Extensive metabolic and biomechanical stress, such as changed membrane tension, cytoskeletal remodelling, oxidative damage, and the accumulation of advanced glycation end products, are caused to chronic hyperglycemia (Gupta et al., 2025[102]). Peripheral nerve degeneration is ultimately triggered by these cascades, which also promote oxidative stress, mitochondrial dysfunction, synaptic impairment, and apoptosis. Increased PIEZO1 activity in dorsal root ganglion neurons increases neuronal excitability and membrane depolarization, both of which are characteristic of neuropathic pain (Yu et al., 2025[262]). This increased excitability amplifies nociceptive signaling and contributes to mechanical allodynia and hyperalgesia observed in DN (Lee et al., 2024[150]). Schwann cell function is also significantly impacted by PIEZO1 dysregulation. Aberrant mechanotransduction via PIEZO1 disrupts myelin maintenance and cytoskeletal architecture in diabetics. Persistent peripheral nerve damage is exacerbated by impaired Schwann cell activity, which alters axonal support, impairs nerve conduction, and restricts regenerative ability (Acheta et al., 2022[2]).

Long-term PIEZO1 overactivation also causes the endoplasmic reticulum calcium homeostasis to be disturbed, which leads to ER stress and the unfolded protein response, both of which accelerate neurodegenerative processes. Vascular dysfunction is another significant effect of PIEZO1 signaling disruption (Wang et al., 2016[244]). In response to shear stress, PIEZO1 normally triggers the PI3K-Akt-eNOS pathway in endothelial cells, encouraging the production of nitric oxide and preserving vascular homeostasis (Qu et al., 2023[194]). By restricting vasodilation, decreasing nitric oxide availability, and compromising endothelial signaling, hyperglycemia hinders this protective function. These alterations worsen neuropathic damage and cause ischemia by decreasing the microvascular perfusion of peripheral nerves. Additionally, PIEZO1 contributes to maintaining the integrity of the blood-nerve barrier; its dysregulation exacerbates neurovascular coupling in diabetes. PIEZO1, which regulates mechanosensitive inflammatory responses, is expressed by T cells, macrophages, and microglia (Zhang et al., 2024[276], Tabrizi et al., 2025[222]). Excessive PIEZO1 activation in diabetes induces pro-inflammatory mediators, including increased generation of reactive oxygen species and cytokines such as TNF-α, IL-6, and IL-1β. Both peripheral and central sensitization are maintained by these inflammatory mediators, which exacerbate neuronal damage. Chronic neuroinflammation and pain persistence are further reinforced in microglia by PIEZO1-driven activation of stress-related pathways, including JNK and mTOR. In general, PIEZO1 has two functions in diabetic neuropathy (Liu et al., 2021[157], Zhang et al., 2024[276], 2025[277]). While healthy PIEZO1 activity preserves neurovascular integrity and mechanosensory function, chronic hyperglycemia results in PIEZO1 maladaptive overactivation. This creates a vicious cycle where mechanical stress and metabolic damage worsen calcium overload, oxidative damage, inflammation, and vascular dysfunction. Experimental results demonstrating that PIEZO1 inhibition decreases neuronal damage and neuropathic pain highlight the importance of PIEZO1 as a possible therapeutic target in diabetic neuropathy (Velasco‐Estevez et al., 2020[235], Shin et al., 2023[211]).

#### PIEZO2

PIEZO2 is a mechanically gated, non-selective cation channel that is genetically required for normal touch sensation and mechanotransduction in mammals. It is predominantly expressed in primary sensory neurons of the dorsal root ganglia, including low-threshold mechanoreceptors, proprioceptors, and subsets of nociceptors. PIEZO2 is also detected in peripheral afferent terminals, Schwann cells, satellite glial cells, and vascular endothelial cells, indicating its presence along the peripheral sensory pathway and neurovascular unit (Ranade et al., 2014[195], Shin et al., 2021[212]).

Under physiological conditions, PIEZO2 channels transduce mechanical forces such as pressure, stretch, and vibration into rapidly adapting inward currents mediated by Na⁺ and Ca²⁺ influx (Lacroix and Wijerathne, 2025[146]). Since most PIEZO2 channels are closed at normal negative resting membrane potentials and only become amenable for mechanical activation after depolarization, channel activity is strictly controlled by membrane voltage (Sánchez-Carranza et al., 2024[205]). By preventing excessive activation by harmless stimuli, this voltage-block mechanism keeps mechanical thresholds of nociceptor high. Membrane depolarization, inflammatory signaling, and modified intracellular second-messenger pathways are among the molecular and functional alterations that dorsal root ganglion neurons experience in neuropathic pain conditions, such as diabetic neuropathy (Garcia-Mesa et al., 2023[84], Fernández-Trillo et al., 2024[73]). Increased cAMP signaling leads to activation of Epac1, a cAMP-dependent exchange protein, which potentiates PIEZO2-mediated mechanotransduction. Increased cAMP signaling leads to activation of Epac1, a cAMP-dependent exchange protein, which potentiates PIEZO2-mediated mechanotransduction. Epac1 depends on cytoskeletal integrity and preferentially increases mechanically triggered PIEZO2 currents in sensory neurons without changing electrical excitability (Garcia-Mesa et al., 2023[84]). Mechanical allodynia and increased PIEZO2 activity are correlated with upregulated Epac1 expression in dorsal root ganglia during neuropathic pain. Relieving the voltage block of PIEZO2 reduces mechanical activation thresholds and promotes continuous activity in nociceptors, according to experimental research (Eijkelkamp et al., 2013[65]). Mechanosensitive currents in Aδ- and C-fiber nociceptors are increased by gain-of-function changes in PIEZO2, leading to a marked hypersensitivity to mechanical stimuli. These results suggest that nociceptor sensitization can be triggered by membrane depolarization brought on by noxious or sensitizing circumstances, which increases the availability of PIEZO2 channels (Sánchez-Carranza et al., 2024[205]). In diabetic distal symmetric polyneuropathy, PIEZO2 expression is increased in cutaneous microvessels, particularly in patients with painful neuropathy. Structural disorganization, endothelial dysfunction, and impaired vasodilation are associated with increased vascular PIEZO2 immunoreactivity. These vascular alterations are proposed to contribute to impaired blood flow and pain severity, linking mechanotransduction dysfunction to microvascular pathology in diabetic neuropathy (Garcia-Mesa et al., 2023[84]). Furthermore, Schwann cells and peripheral glial components express PIEZO2, and several inflammatory and neuropathic pain models have been shown to exhibit PIEZO2 overexpression (Wan et al., 2024[239]). While increasing PIEZO2 activity has been correlated with sensitivity to typically harmless mechanical stimuli, loss of function or knockdown of PIEZO2 lowers mechanical allodynia (Nencini et al., 2021[181]). All of these results point to PIEZO2's involvement in aberrant mechanotransduction, nociceptor sensitization, and neurovascular dysfunction in the formation and maintenance of mechanical pain in neuropathic dysfunction.

## Current Ion Channel-Targeted Therapies

Therapeutic strategies targeting ion channels in painful DN have evolved significantly, motivated by the recognition that sensory neuron hyper-excitability in diabetes is not just a structural consequence of nerve injury but is fundamentally driven by maladaptive ion‐channel plasticity. In PDN, VGSCs, VGCCs, potassium (K⁺) channels, TRP channels, purinergic (P2X/P2Y) receptors and mechanosensitive PIEZO channels each contribute to the aberrant excitability, spontaneous firing and enhanced nociceptive transmission characteristic of the condition. The pharmacological aim is therefore to modulate or normalise channel dysfunction to alleviate pain. Several classes of agents are either already in clinical use or in preclinical/clinical development, yet many translational hurdles remain.

Among the Na_v_ channels, Na_v_1.7, Na_v_1.8 and Na_v_1.9 are the most strongly implicated in PDN. Na_v_1.7 is consistently upregulated in dorsal root ganglion (DRG) neurons from diabetic rodents, promoting exaggerated firing; selective Na_v_1.7 blockers such as PF-05089771, and vixotrigine (BIIB074), BIIB-095 reduce hyperexcitability in preclinical neuropathy and have progressed into Phase II trials in neuropathic pain (Kingwell, 2019[138], Bigsby et al., 2022[23]). Na_v_1.8, which mediates repetitive firing in nociceptors, contributes to mechanical hypersensitivity in experimental diabetes, and inhibitors such as VX-150, VX-548 (suzetrigine), A-803467, or even the repurposed drug ambroxol demonstrate robust antinociceptive efficacy in diabetic rats (Witty et al., 2017[251], Vaelli et al., 2024[232]). Na_v_1.9, characterised by a persistent subthreshold current, also sustains diabetic neuronal hyperexcitability; silencing Na_v_1.9 using siRNA significantly reduces spontaneous DRG firing in diabetic models (Huang et al., 2014[117]). Although these results point to strong mechanistic rationale, no Na_v _isoform-selective therapy is yet approved for PDN, largely due to safety concerns, compensatory channel changes, and translational gaps between rodent and human channel expression (Skerratt and West, 2015[214]).

VGCCs, particularly Ca_v_2.2 (N-type) and Ca_v_3.2 (T-type) channels, represent another well-characterised therapeutic axis. Ca_v_2.2 mediates neurotransmitter release at nociceptive synapses, and its blockade by ziconotide, a synthetic ω-conotoxin MVIIA, provides strong analgesia in refractory neuropathic pain, though intrathecal administration limits its use. Small-molecule N-type blockers such as TROX-1 show benefit in preclinical PDN (McGivern, 2007[172], Abbadie et al., 2010[1]). Ca_v_3.2, a T-type channel central to subthreshold oscillations, is upregulated in DRG neurons in diabetes; selective T-type antagonists such as TTA-P2 and ethosuximide reverse mechanical allodynia in STZ-diabetic rodents (Todorovic and Jevtovic-Todorovic, 2011[227]). Clinically, the most established VGCC-targeting agents are the α₂δ ligands gabapentin and pregabalin, which bind the auxiliary α₂δ-1 subunit that is itself upregulated in diabetic nerves, thereby reducing presynaptic calcium entry; these remain first-line PDN therapies with validated efficacy (Jang and Oh, 2023[124]). While VGCC-targeted therapies have achieved the greatest clinical penetration among ion-channel drugs, side effects (e.g., dizziness, sedation) and incomplete relief in many patients reveal the need for more selective, peripherally restricted agents.

Potassium channels, particularly KCNQ2/3 (M-channels), Kᵥ1.2, and Kir6.2, play a critical role in stabilising membrane potential and providing repolarising drive, yet are downregulated or functionally impaired in PDN. KCNQ2/3 downregulation leads to heightened excitability, and KCNQ openers such as retigabine (ezogabine) and flupirtine robustly reverse hyperalgesia in diabetic rodent models, though retigabine's human use was discontinued due to pigmentation toxicity (Wu et al., 2025[252]). Kᵥ1.2 reduction in DRG neurons disrupts rapid repolarisation; experimental Kᵥ1.2 openers (4-AP analogues) restore excitability balance in preclinical studies (Zhang et al., 2021[272]). Kir6.2, part of the ATP-sensitive K_ATP_ channel family, is metabolically dysregulated in hyperglycaemia; openers such as diazoxide and nicorandil enhance hyperpolarising currents and attenuate diabetic pain in experimental models (Nakai-Shimoda et al., 2022[180]). Potassium-channel modulation holds promise as a means of reinstating lost inhibitory tone, but specificity and safety (especially cardiovascular effects) require further refinement.

The TRP family, including TRPV1, TRPA1, and TRPM8, is deeply involved in PDN because diabetic metabolic stress sensitises these polymodal channels. TRPV1, a major heat sensor, becomes hyperactive in diabetes; the capsaicin 8 % patch is an approved analgesic treatment for PDN, acting through high-dose agonist-induced nociceptor defunctionalisation (Bonezzi et al., 2020[26]). Resiniferatoxin, a TRPV1 superagonist, is another candidate with potent defunctionalising capacity (Baskaran et al., 2023[18]). TRPA1, activated by reactive carbonyl species elevated in diabetic oxidative stress, is effectively inhibited by antagonists such as HC-030031 and A-967079, producing strong reversal of hyperalgesia in diabetic rats (Koivisto et al., 2022[142]). TRPM8, although less studied, contributes to cold allodynia; its agonists (menthol) and antagonists (AMTB) modulate cold hypersensitivity in preclinical PDN (Knowlton et al., 2010[139], Cao et al., 2019[34], Li et al., 2022[153]). TRP-targeted therapeutics present an opportunity to address specific sensory modalities (heat, cold, mechanical hypersensitivity), and may be most effective as part of combination regimens.

Purinergic channels, particularly P2X3, P2X7, and P2Y12, integrate nociceptive and inflammatory signaling and are strongly implicated in PDN. P2X3, an ATP-gated ion channel on nociceptors, contributes to spontaneous activity in diabetic nerves; the selective antagonist gefapixant is already in Phase III trials for chronic cough and shows promising preclinical PDN benefits (Richards et al., 2019[199], Sharma et al., 2024[208]). P2X7 receptors on microglia mediate IL-1β release and neuroinflammation; antagonists such as Brilliant Blue G and AZD9056 reduce diabetic neuroinflammatory pain in vivo (Wang et al., 2020[241], Ren and Illes, 2022[197], Liu et al., 2023[159]). P2Y12, a microglial metabotropic receptor, can be antagonised by clopidogrel or PSB-0739, both of which show attenuation of diabetic pain in preclinical models (Tozaki-Saitoh et al., 2008[229], Zhang et al., 2023[274]). Purinergic targeting is particularly attractive in early inflammatory or metabolically active phases of PDN, though specificity remains a major challenge due to ATP's diverse physiological roles.

Mechanosensitive PIEZO1 and PIEZO2 channels constitute one of the most novel therapeutic areas in PDN. Diabetes induces oxidative and metabolic sensitisation of PIEZO channels, enhancing mechanical allodynia. Inhibiting PIEZO1 with the peptide GsMTx4 significantly reduces mechanical hypersensitivity in diabetic rodents (Liu et al., 2024[158], Gupta et al., 2025[102]). PIEZO2, which governs tactile and proprioceptive signaling, also contributes to diabetic mechanical allodynia, and gene-silencing strategies using siRNA reduce aberrant mechanosensory firing (Murthy et al., 2018[179], Sánchez-Carranza et al., 2024[205]). PIEZO-targeted therapies remain entirely preclinical but represent a mechanistically unique approach, modulating pressure-transduction pathways that are directly altered in diabetic nerve pathology.

The current landscape demonstrates that several ion-channel modulators, from sodium and calcium channel blockers to potassium channel openers, TRP antagonists, purinergic inhibitors and PIEZO regulators, have substantial mechanistic and preclinical support, with a few already in clinical practice (gabapentinoids, capsaicin patch, ziconotide). Despite this progress, clinical translation remains hindered by limited selectivity, compensatory plasticity, central side effects, and patient heterogeneity. Only a fraction of patients achieve meaningful relief with current therapies (Jang and Oh, 2023[124]), reinforcing the need for more precise, peripherally targeted and combination approaches.

See also Table 1(Tab. 1) (References in Table 1: Abbadie et al., 2010[1]; Baskaran et al., 2023[18]; Bigsby et al., 2022[23]; Bonezzi et al., 2020[26]; Cao et al., 2019[34]; Huang et al., 2014[117]; Jang and Oh, 2023[124]; Kingwell, 2019[138]; Knowlton et al., 2010[139]; Koivisto et al., 2022[142]; Li et al., 2022[153]; Liu et al., 2023[159]; Liu et al., 2024[158]; McGivern, 2007[172]; Murthy et al., 2018[179]; Nakai-Shimoda et al., 2022[180]; Ren and Illes, 2022[197]; Richards et al., 2019[199]; Sharma et al., 2024[208]; Todorovic and Jevtovic-Todorovic, 2011[227]; Tozaki-Saitoh et al., 2008[229]; Vaelli et al., 2024[232]; Wang et al., 2020[241]; Witty et al., 2017[251]; Wu et al., 2025[252]; Zhang et al., 2021[272]; Zhang et al., 2023[274])

## Challenges and Future Directions

Despite major advances in delineating ion-channel dysfunction in painful diabetic neuropathy (PDN), substantial conceptual, translational, and clinical barriers continue to impede therapeutic progress. A central challenge lies in the inherent complexity and heterogeneity of PDN pathophysiology. Rather than representing a linear consequence of hyperglycaemia, PDN emerges from an integrated network of metabolic stress, mitochondrial impairment, microvascular deficits, immune-glial activation, lipid dysregulation, and oxidative injury, all of which converge to drive ion-channel plasticity and nociceptor hyperexcitability (Joksimovic et al., 2022[128] , Wang et al., 2024[243], Yang et al., 2025[259]). This multifactorial landscape results in multiple pathological entry points; consequently, selective blockade of a single ion-channel subtype often yields incomplete benefit, as compensatory mechanisms such as Naᵥ isoform switching, T-type Ca²⁺ channel upregulation, and TRP sensitisation rapidly restore aberrant excitability (Duzhyy et al., 2015[62], Pabbidi and Premkumar, 2017[186], Bigsby et al., 2022[23]).

Translational limitations of preclinical models add an additional layer of complexity. STZ-induced and genetic (db/db) rodent models replicate metabolic dysregulation but fail to fully capture the chronicity, sensory heterogeneity, and comorbidity patterns typical of human PDN (O'Brien et al., 2014[185], Pham et al., 2019[189]). Moreover, species differences in DRG ion-channel expression, particularly for Naᵥ1.7, Naᵥ1.8, TRPA1, and P2X3, undermine predictive validity, contributing to repeated translational failures of ion-channel modulators in clinical trials (Chen and Kym, 2009[42], Serrano et al., 2012[207], Skerratt and West, 2015[214], Chang et al., 2018[41]). These discrepancies underscore the need for human-relevant platforms, such as iPSC-derived sensory neurons and ex-vivo human DRG preparations.

Clinical heterogeneity further complicates therapeutic targeting. PDN encompasses diverse sensory phenotypes, burning pain, cold allodynia, mechanical hypersensitivity, and paroxysmal electric-shock pain, each underpinned by distinct molecular signatures (Tesfaye et al., 2013[224], Themistocleous et al., 2016[225]). Although gain-of-function mutations in SCN9A (Naᵥ1.7) or SCN10A (Naᵥ1.8) can drive hyperexcitability in a minority of patients, these variants account for <20 % of painful neuropathy cases, highlighting the need for phenotype-guided or biomarker-guided enrolment in clinical trials (Faber et al., 2012[67], Bennett, 2014[21]). Failure to match molecular pathology with the channel-targeted intervention has likely contributed to modest efficacy outcomes in several Phase II/III studies.

Safety and off-target toxicity represent additional barriers, given the ubiquitous physiological roles of ion channels in cardiac, CNS, vascular and endocrine systems. Sodium-channel blockers risk arrhythmias and cognitive effects, whereas calcium-channel modulators influence autonomic and cardiovascular function (Eijkelkamp et al., 2012[66], Priest and McDermott, 2015[192], Huang et al., 2017[116]). TRPV1 antagonists, despite strong mechanistic rationale, consistently produced marked hyperthermia in early trials, ultimately limiting systemic deployment (Garami et al., 2018[82]). Strategies such as peripherally restricted molecules, nanoparticle-mediated nerve-targeting, and transdermal or microneedle delivery may mitigate such toxicities but remain largely experimental.

Therapeutic timing is another critical yet underexplored dimension. Ion-channel dysregulation arises early in diabetes, preceding structural axonal degeneration and central sensitisation (Feldman et al., 2019[70], Eid et al., 2023[64]). Once irreversible small-fibre loss, sustained microglial activation, and dorsal horn remodeling are established, functional modulation of ion-channels may confer only limited benefit. Nevertheless, most clinical trials recruit individuals with chronic PDN, inherently reducing therapeutic responsiveness (Calcutt, 2020[32], Kalteniece et al., 2020[132], Pop-Busui et al., 2022[190]). Earlier intervention, potentially even at pre-symptomatic stages, may therefore be essential.

A further barrier is the absence of validated biomarkers that reflect ion-channel dysfunction. Although nerve excitability indices, intra-epidermal nerve fibre (IENF) density, corneal confocal microscopy, DRG imaging, and circulating microRNAs show promise, none have yet achieved sufficient standardisation for clinical application (Ismail, 2023[120], Tavakoli et al., 2023[223]). This lack of mechanistic biomarkers prevents precision selection of patients most likely to benefit from Naᵥ-, Caᵥ-, TRP- or purinergic-targeted therapies.

Clinical trial design and regulatory constraints add to these challenges. Neuropathic pain trials are characterised by high placebo response rates, often >30 % and outcome variability, complicating detection of drug effects (Freeman et al., 2015[78]). Ion-channel modulators may introduce temperature-related or sensory-specific side-effects, raising the risk of functional unblinding. Regulatory agencies also require extensive long-term cardiac and neurological safety monitoring, prolonging development timelines and intensifying costs, particularly for combination therapies (Waszkielewicz et al., 2013[246], Garami et al., 2020[83], Felix et al., 2025[72]).

Looking ahead, meaningful progress will require integrated strategies that combine molecular precision, translational fidelity and innovative delivery platforms. Mechanistic phenotyping using skin-biopsy transcriptomics, single-cell DRG atlases, quantitative sensory testing, and high-resolution nerve excitability profiling could enable channel-specific patient stratification (Marshall et al., 2021[169], Guo et al., 2024[98], Lee and Won, 2025[147]). Rational multi-target approaches, such as concurrent Naᵥ1.7 blockade with KCNQ activation, or TRPA1 inhibition paired with anti-inflammatory or mitochondrial-stabilising agents, may overcome compensatory mechanisms and produce synergistic analgesia (Koivisto et al., 2012[141], Alles and Smith, 2021[4], Wang et al., 2024[243], Zhang et al., 2025[270]). Human-relevant models, including iPSC-derived nociceptors and organ-on-chip systems, will be essential for refining target validation (Labau et al., 2022[145], Zhu et al., 2025[283]). Finally, adaptive trial designs, biomarker-enriched cohorts, and patient-centred outcome measures may accelerate the translation of ion-channel therapeutics while ensuring clinical relevance.

## Discussion & Conclusion

Painful diabetic neuropathy (PDN) represents one of the most persistent and disabling complications of diabetes mellitus, affecting up to 50 % of long-standing diabetic patients, with 15-25 % experiencing chronic neuropathic pain that profoundly impairs quality of life (Jang and Oh, 2023[124], Gupta et al., 2025[103] ). Despite significant advancements in glycemic control and pharmacotherapy, effective and sustained relief from PDN remains elusive. This clinical gap reflects the multifactorial nature of PDN, involving a cascade of metabolic, inflammatory, and neurodegenerative mechanisms leading to peripheral nerve dysfunction (Abbadie et al., 2010[1], Ismail, 2023[120]). Among the multitude of cellular players involved, ion channels have emerged as central regulators of sensory neuronal excitability and plasticity. Aberrant ion channel expression, altered gating kinetics, and disrupted trafficking contribute to the hyperexcitability of nociceptors and spontaneous ectopic discharges characteristic of neuropathic pain (Trimmer, 2014[230], Joksimovic et al., 2022[128]).

The past decade has seen increasing recognition of the critical roles played by distinct ion channel families in PDN pathophysiology, including voltage-gated sodium (Naᵥ) channels, calcium (Caᵥ) channels, potassium (Kᵥ) channels, transient receptor potential (TRP) channels, purinergic P2X/P2Y receptors, and mechanosensitive PIEZO channels. Each ion channel contributes uniquely to the altered electrical landscape of diabetic sensory neurons. For instance, upregulation of Naᵥ1.7, Naᵥ1.8, and Naᵥ1.9 channels enhances depolarization and abnormal firing in primary afferents (Bigsby et al., 2022[23]), while reduced expression of KCNQ (Kᵥ7) and inwardly rectifying potassium (Kir) channels diminishes repolarizing currents, prolonging action potential (Djouhri et al., 2020[55]). Calcium channel dysfunction, especially involving N-type and T-type VGCCs, facilitates excessive neurotransmitter release from nociceptive terminals and amplifies central sensitization (Harding and Zamponi, 2022[106]). TRP channels such as TRPV1, TRPA1, and TRPM8 further integrate thermal, oxidative, and chemical stimuli, translating metabolic stress into pain signals (Pabbidi and Premkumar, 2017[186]). Similarly, ATP-gated P2X3 and P2X7 receptors on neurons and glia sustain neuroinflammation (Wang et al., 2024[243] ), while PIEZO1 and PIEZO2 channels contribute to aberrant mechanotransduction and tactile allodynia (Wan et al., 2024[239], Gupta et al., 2025[102]).

Despite this mechanistic understanding, the clinical translation of ion channel modulators has been fraught with challenges. Approved drugs such as pregabalin and gabapentin, α2δ ligands that indirectly reduce presynaptic Ca²⁺ influx, remain first-line therapies, but only 30-40 % of patients achieve meaningful relief, and side effects like sedation or dependence limit long-term use (Azmi et al., 2019[14]). Sodium channel blockers such as carbamazepine, oxcarbazepine, and lacosamide exhibit partial efficacy in subsets of patients, yet their narrow therapeutic window and cardiac safety issues restrict broader application (Alsaloum et al., 2025[5]). While TRPV1 antagonists initially appeared promising, clinical trials were halted due to hyperthermia and loss of heat sensation (Liu et al., 2023[156]). Similarly, efforts to develop selective Naᵥ1.7 inhibitors, such as vixotrigine and funapide, yielded mixed outcomes, highlighting the complexity of compensatory ion channel expression in chronic neuropathy (Witty et al., 2020[250], Dormer et al., 2023[58]).

A major barrier to progress lies in the inherent redundancy and plasticity of nociceptive ion channel networks (Joksimovic et al., 2022[128]). Chronic hyperglycemia and oxidative stress alter not one, but multiple channel types simultaneously, leading to widespread electrophysiological reprogramming (Wang et al., 2024[243]). Therefore, selective blockade of a single channel subtype often fails to reverse the pain phenotype entirely. In this context, recent preclinical and translational evidence supports the notion that multi-target or combination strategies may yield superior outcomes. For example, concurrent targeting of Naᵥ and Kᵥ channels with novel compounds such as E0199 restores a more physiological balance between excitatory and inhibitory conductances (Zhang et al., 2025[270]), while combining TRPA1 antagonists with antioxidants mitigates oxidative stress-induced hyperexcitability (Fila et al., 2024[75]). 

These findings collectively point toward the necessity of an integrated therapeutic paradigm, one that transcends the limitations of single-pathway inhibition and addresses the interconnected molecular cascades underpinning PDN. Future pharmacological strategies could leverage polypharmacology and network pharmacology approaches, wherein drugs or drug combinations are rationally designed to engage multiple ion channel subtypes and auxiliary targets involved in oxidative stress, mitochondrial dysfunction, and neuroinflammation (Joksimovic et al., 2022[128], Jin et al., 2025[126]). The emergence of dual-functional molecules, such as compounds that simultaneously block Naᵥ and open Kᵥ channels (Zhang et al., 2025[270]), for example E0199, exemplifies this shift. Additionally, nanocarrier-based co-delivery systems enable spatiotemporally controlled release of multiple ion channel inhibitors, improving bioavailability and reducing systemic toxicity (Lee and Yeo, 2015[148]). Gene therapies also hold potential, for instance, siRNA-mediated Naᵥ knockdown combined with KCNQ overexpression may normalize neuronal excitability and improve conduction velocity in diabetic models.

Another promising avenue lies in precision medicine. Interindividual differences in ion channel gene variants (e.g., SCN9A, KCNN2, TRPA1 polymorphisms) modulate susceptibility to PDN and responsiveness to channel-targeted drugs. Integration of genomic, proteomic, and electrophysiological profiling could facilitate patient stratification and personalized treatment regimens (Ślęczkowska et al., 2022[215], Khan et al., 2025[136]). The growing availability of induced pluripotent stem cell (iPSC), derived sensory neuron models and organoids offers unprecedented opportunities to model PDN pathophysiology in vitro, screen ion channel modulators, and predict clinical efficacy (Yang et al., 2019[256], Van Lent et al., 2024[233], Lee and Won, 2025[147]). 

Nevertheless, substantial obstacles remain for considering ion channels as primary drug targets. Ion channels are widely expressed across excitable and non-excitable tissues, raising the risk of off-target cardiac, skeletal, and autonomic effects. Drug development must therefore emphasize selectivity and tissue specificity, possibly via targeted delivery systems or allosteric modulators that preferentially act on pain-related isoforms (Kaczorowski et al., 2008[130], Gerlach and Antonio, 2015[87]). Another critical limitation lies in the poor predictive validity of current animal models. While STZ-induced diabetic rodents replicate many metabolic and electrophysiological hallmarks of PDN, they inadequately reflect the chronicity and comorbidities of human disease (Islam, 2013[119], Goyal et al., 2016[94]). Translation to clinical benefit will require not only improved models but also more robust biomarkers, such as skin nerve fiber density, microneurography, or circulating inflammatory markers, to objectively assess treatment response (Fan and Gordon Smith, 2022[69]).

From a translational perspective, combination therapies incorporating metabolic modulators (e.g., α-lipoic acid, benfotiamine), anti-inflammatory agents (minocycline, curcumin), and ion channel-targeted drugs could address both upstream and downstream contributors to neuronal hyperexcitability. Early-phase clinical trials investigating such integrated approaches are promising but warrant validation in larger cohorts. Importantly, future interventions should aim not merely to suppress pain but also to promote neuroprotection and regeneration, as reversal of small fiber loss is achievable with optimal control of excitotoxicity and oxidative damage.

In conclusion, the last two decades of research have firmly established ion channels as indispensable mediators of PDN pathogenesis and as viable therapeutic targets. The challenge now lies in translating this mechanistic insight into safe, effective, and durable therapies. Integrative strategies that combine selective ion channel modulation with metabolic and anti-inflammatory correction, guided by patient-specific molecular profiles, represent the most rational path forward. The convergence of electrophysiology, molecular pharmacology, and systems biology promises to transform the therapeutic landscape of PDN, moving beyond symptomatic relief toward genuine disease modification. Continued interdisciplinary collaboration between basic scientists, pharmacologists, and clinicians will be essential to harness the full potential of ion channel-based therapeutics and ultimately improve outcomes for patients suffering from this pervasive and intractable complication of diabetes.

## Declaration

### Author Contributions

**TG:** Conceptualization, Designing original draft, Writing original draft- lead, Editing- lead & Compilation. **AA:** Writing- support, Editing- support. **RC: **Writing- support. **AK: **Writing- support.** TGS: **Reviewing & Finalising. **RS: **Conceptualization, Supervision, Reviewing & Finalising. All authors read and agreed with the final version of the manuscript prior to submission.

### AI Disclosure

Authors confirm that we have used ChatGPT and Grammarly to assist in improving the language and readability of our manuscript. After its use, we thoroughly reviewed and verified all AI-assisted content to ensure scientific accuracy, originality, and compliance with ethical standards.

### Consent for publication

Not applicable.

### Funding

None.

### Conflict of interest

The authors declare no conflict of interest financial or otherwise.

### Acknowledgements

The authors are grateful to the Department of Science and Technology, New Delhi, for providing DST-INSPIRE Fellowship to Tanya Gupta.

## Figures and Tables

**Table 1:**
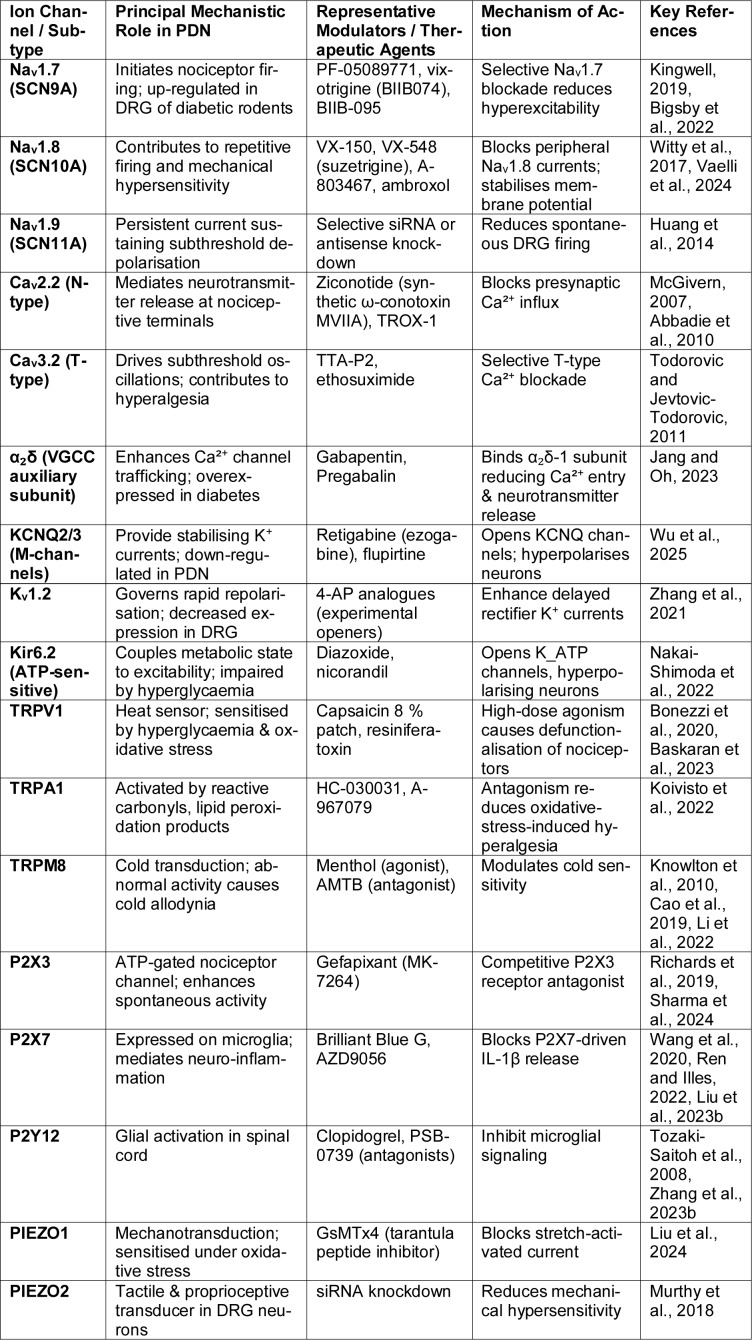


**Figure 1:**
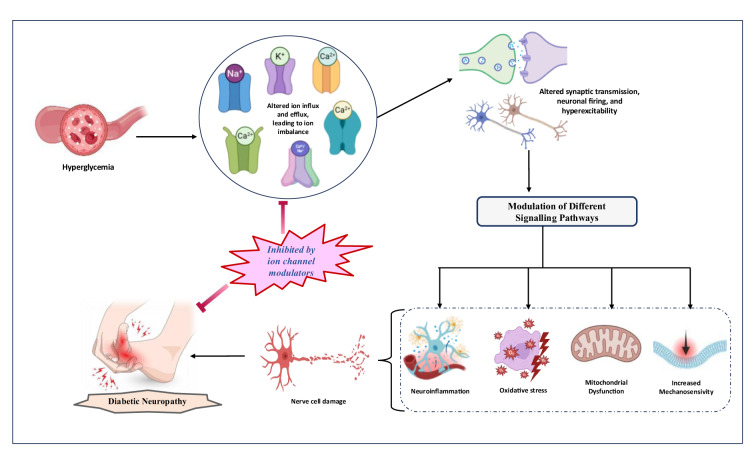
Graphical abstract
